# Proinsulin‐specific T‐cell responses correlate with estimated c‐peptide and predict partial remission duration in type 1 diabetes

**DOI:** 10.1002/cti2.1315

**Published:** 2021-07-26

**Authors:** Yassmin Musthaffa, Emma E Hamilton‐Williams, Hendrik J Nel, Anne‐Sophie Bergot, Ahmed M Mehdi, Mark Harris, Ranjeny Thomas

**Affiliations:** ^1^ Department of Endocrinology and Diabetes Queensland Children’s Hospital South Brisbane QLD Australia; ^2^ The University of Queensland Diamantina Institute The University of Queensland Brisbane QLD Australia

**Keywords:** antigens, CD4^+^ T cells, Islet epitopes, proinsulin, proliferation, type 1 diabetes

## Abstract

**Objective:**

Type 1 diabetes (T1D) is an autoimmune disorder in which autoreactive T cells destroy insulin‐producing β‐cells. Interventions that preserve β‐cell function represent a fundamental therapeutic goal in T1D and biomarkers that predict and monitor β‐cell function, and changes in islet autoantigenic signatures are needed. As proinsulin and neoantigens derived from proinsulin peptides (hybrid insulin peptides, HIPs) are important T1D autoantigens, we analysed peripheral blood CD4^+^ T‐cell autoantigen‐specific proliferative responses and their relationship to estimated β‐cell function.

**Methods:**

We recruited 72 people with and 42 without T1D, including 17 pre‐diabetic islet antibody‐positive and 9 antibody‐negative first‐degree relatives and 16 unrelated healthy controls with T1D‐risk HLA types. We estimated C‐peptide level at 3‐month intervals for 2 years post‐diagnosis and measured CD4^+^ T‐cell proliferation to proinsulin epitopes and HIPs using an optimised bioassay.

**Results:**

We show that CD4^+^ T‐cell proliferation to any islet peptide and to multiple epitopes were significantly more frequent in pre‐diabetic islet antibody‐positive siblings and participants with T1D ≤ 3 months of duration, than in participants with T1D > 3 months or healthy controls. Among participants with T1D and first‐degree relatives, CD4^+^ T‐cell proliferation occurred most frequently in response to proinsulin_33‐63_ (full‐length C‐peptide). Proinsulin_33‐63_‐specific responses were associated with *HLA‐DR3‐DQ2* and/or *HLA‐DR4/DQ8*. In children with T1D, proinsulin_33‐63_‐specific T‐cell proliferation positively associated with concurrent estimated C‐peptide and predicted survival in honeymoon.

**Conclusion:**

CD4^+^ T‐cell proliferative responses to proinsulin‐containing autoantigens are common before and immediately after diagnosis of T1D but decline thereafter. Proinsulin_33‐63_‐specific CD4^+^ T‐cell response is a novel marker of estimated residual endogenous β‐cell function and predicts a better 2‐year disease outcome.

## Graphical abstract

In this study, we found CD4^+^ T‐cell proliferative responses to proinsulin‐containing autoantigens are common before and immediately after diagnosis of type 1 diabetes but decline thereafter. Proinsulin_33‐63_‐specific CD4^+^ T‐cell response is a novel marker that correlates with increased estimated endogenous insulin production.

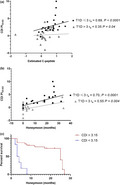

## Introduction

Type 1 diabetes (T1D) is a chronic, incurable autoimmune disorder in which insulin‐producing β cells are destroyed by islet‐infiltrating T cells.^1^, ^2^ Before and after clinical onset, pancreatic islets undergo β‐cell loss. Despite recent advances in disease management tools, the medical and psychological burden on children and their families is high, and diabetic complications including hypoglycaemia and microvascular disease are common. There has been progress towards understanding the genetic, environmental and immunologic basis for TID^3^; however, prevention and/or cure of this condition remain elusive. The clear benefits of preserving endogenous insulin secretion include a longer honeymoon period, better metabolic control and fewer complications.^4^, ^5^ Moreover, improved metabolic control early in the course of T1D may be protective by producing a ‘metabolic memory’.^4^, ^6^ Therefore, interventions that preserve β‐cell function in T1D represent a fundamental therapeutic goal. Indeed, many patients have detectable β‐cell function for many years after diagnosis,^7^ and immune intervention may be capable of preserving a significant proportion of β‐cells.^8^

The rate of decline of β‐cell function is heterogeneous,^9^ and the factors that preserve β‐cell function are poorly understood. Furthermore, the response to immune interventions also varies amongst individuals. Robust immune biomarkers to predict and monitor β‐cell function are needed, to assess the short‐term impact of immune interventions, with the aim of improving longer‐term metabolic outcome in T1D. Islet‐reactive CD4^+^ and CD8^+^ T cells play a central role in the pathogenesis of β‐cell destruction. For CD8^+^ T cells, the balance between β‐cell‐specific stem cell memory and exhaustion contributes to the rate of disease progression and may be skewed towards exhaustion by immunotherapies such as teplizumab. For CD4^+^ T cells, the frequency of peripheral blood (PB) T helper 2‐like CD25^+^CD127^hi^ T cells was recently associated with improved β‐cell function.^10^ Favorable responses to agents such as Alefacept are also seen in individuals with a higher frequency of CD25^+^CD127^hi^ T cells, suggesting that CD127^hi^ cells maintain an anti‐inflammatory environment that supports improved β‐cell function and response to immunotherapy.^10^

Islet‐infiltrating CD4^+^ T cells recognise epitopes derived from the proinsulin C‐peptide, and from neoantigens such as hybrid insulin peptides (HIPs) formed in β‐cells by the fusion of C‐peptide fragments to peptides of chromogranin A, islet amyloid polypeptide or C‐peptide itself.^1^, ^11^, ^12^ Several lines of evidence indicate that insulin or its precursor proinsulin is a primary autoantigenic target of T cells in T1D.^13^, ^14^ In humans, antibodies to insulin are the first marker of pre‐diabetes in genetically at‐risk infants followed from birth, and precede antibodies specific for other islet antigens.^15^ The insulin gene locus is a T1D susceptibility gene,^16^, ^17^ and its regulatory VNTR regions play an essential role in insulin‐specific self‐tolerance.^16^, ^17^, ^18^, ^19^ Insulin expression is restricted to β‐cells of pancreatic islets, whereas other T1D autoantigens are expressed more widely. Post‐mortem analyses of pancreatic tissue of T1D show that insulitis is usually detected in islets with insulin‐positive β‐cells.^20^, ^21^ Accordingly, proinsulin peptides have been successfully used in T‐cell assays^22^, ^23^, ^24^, ^25^ and the ‘C19‐A3’ epitope employed in several early phase immunotherapy trials.^26^, ^27^ Proinsulin is also a primary β‐cell antigen target for autoreactive T cells in spontaneous autoimmune diabetes in the non‐obese diabetic (NOD) mouse model.^28^ Studies in transgenic mice suggest that other β‐cell antigens may subsequently be recognised as a result of epitope spreading.^29^ We hypothesised that circulating CD4^+^ T cells in patients with recent‐onset T1D and AB+ siblings at high risk would respond to specific islet auto‐antigenic peptides. Since epitopes of proinsulin and hybrid islet peptides containing proinsulin sequences have been described,^1^, ^11^, ^12^, ^30^ we measured autoreactive CD4^+^ T‐cell proliferation towards a panel of proinsulin peptides. We used our optimised fit‐for‐purpose CFSE‐based T‐cell proliferation assay,^31^ to evaluate the frequency of positive responses to each epitope, and to determine the relationship between the level of proliferation and the clinical features of the patients with T1D. We found that CD4^+^ T‐cell responses to proinsulin_33‐63_ were the most frequent of the islet peptides tested, and that the level of proinsulin_33‐63_‐specific CD4^+^ T‐cell proliferation was positively associated with β‐cell function and predicted survival in remission, suggesting that proinsulin_33‐63_‐specific CD4^+^ T‐cell proliferation is a novel immune biomarker of predicted β‐cell function.

## RESULTS

### CD4^+^ T‐cell proliferative responses to proinsulin epitopes occur more frequently than in healthy controls, in the pre‐diabetic period and soon after onset of T1D

**Figure 1:**
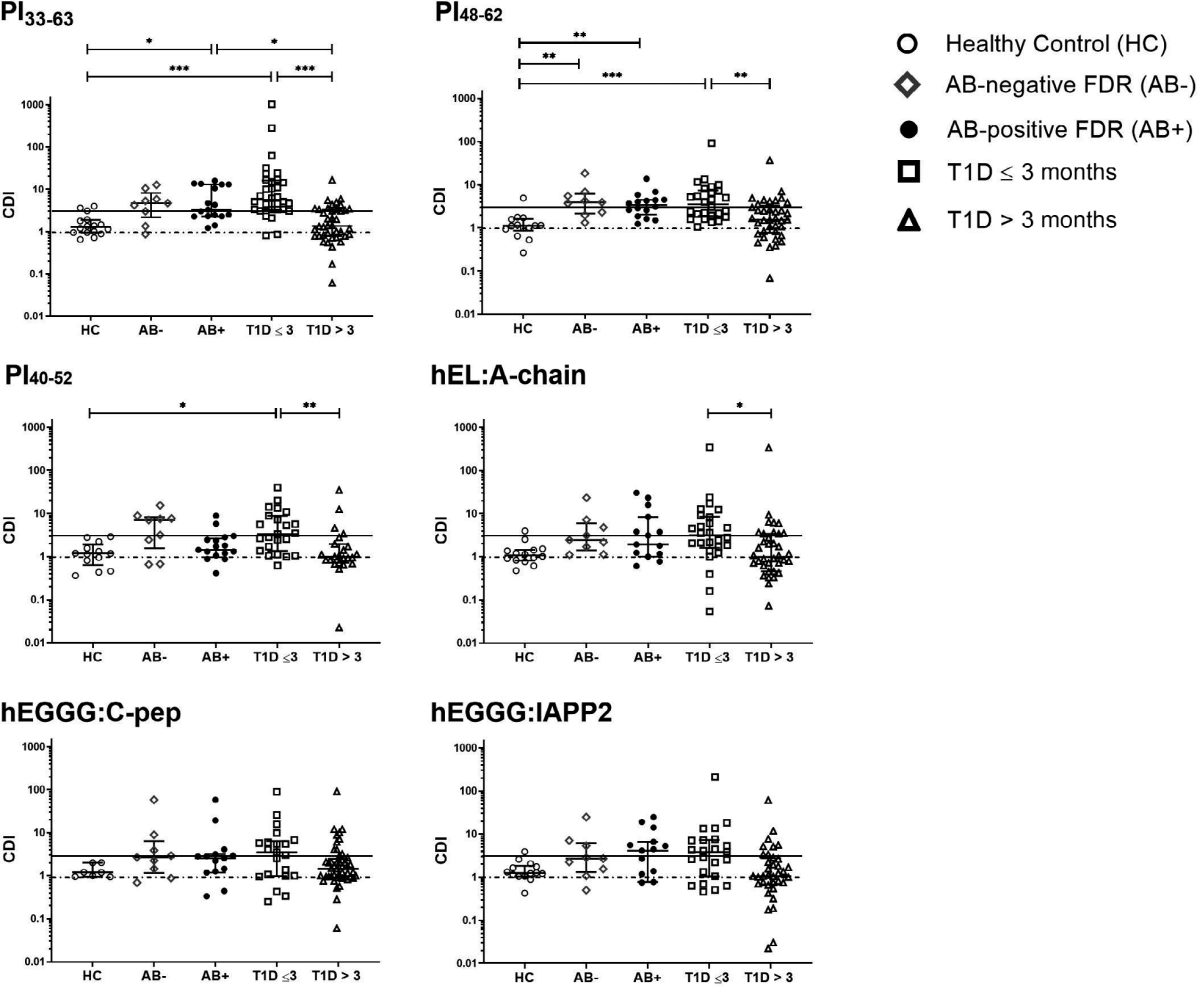
The CD4^+^ T‐cell response to proinsulin but not HIP epitopes is increased in the pre‐diabetes period and after T1D onset. Each point represents an individual CDI calculated as the mean of unstimulated proliferation in three technical replicates for each patient (healthy control, *n* = 16; AB‐negative FDR, *n* = 9, AB‐positive FDR, *n* = 17; T1D ≤ 3 months, *n* = 32; T1D > 3 months, *n* = 40). Error bars display the median ± IQR. *P < 0.05, **P < 0.01, ***P < 0.001. Solid line, CDI = 3. Dashed line, CDI = 1. Kruskal–Wallis post‐hoc Dunn’s test with *P*‐values adjusted via Benjamini–Hochberg. CDI, Cell Division Index.

**Figure 2:**
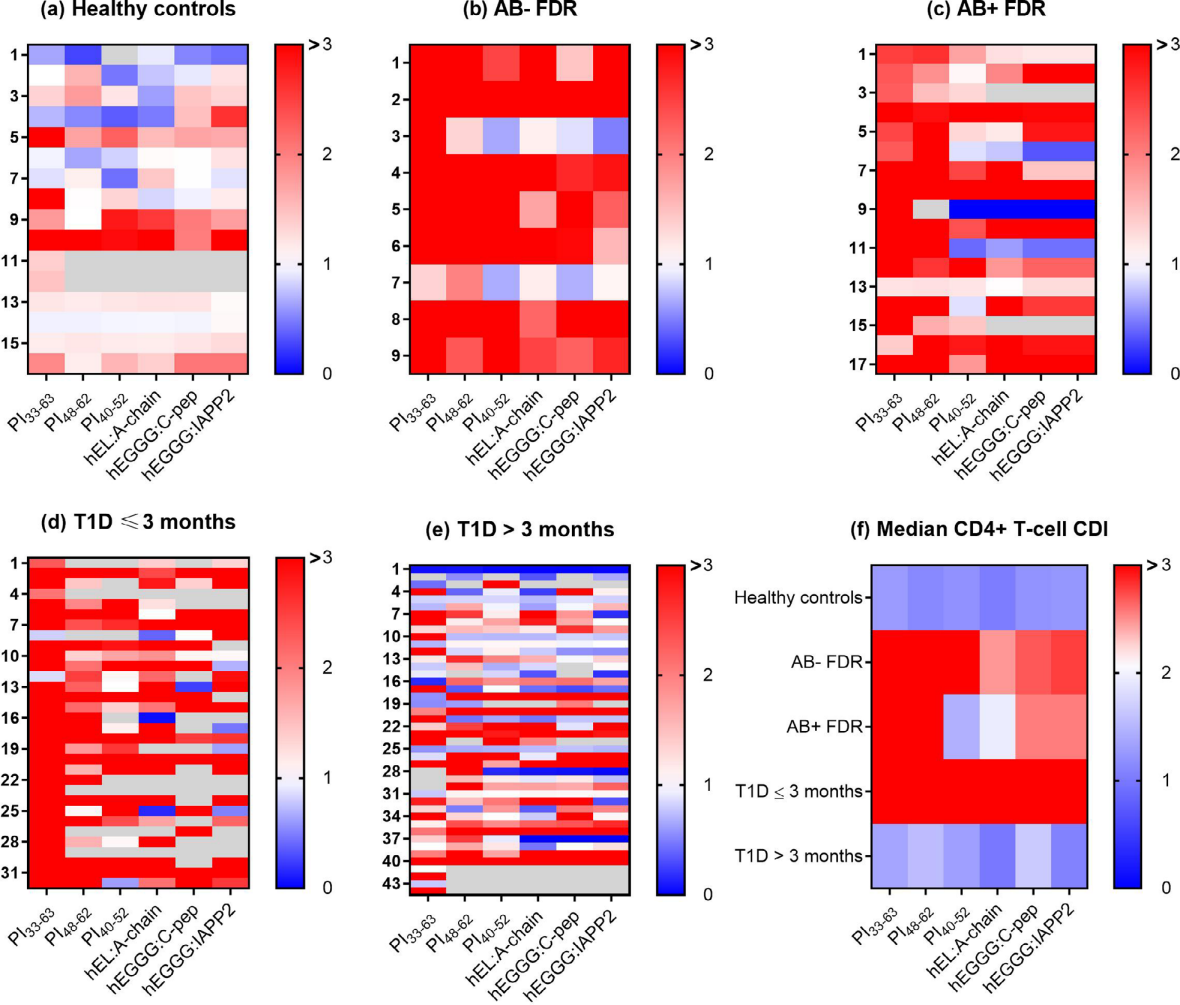
Comparison of antigen response profile between groups. **(a)** Healthy controls, *n* = 16; **(b)** AB‐negative FDR, *n* = 9; **(c)** AB‐positive FDR, *n* = 17; **(d)** T1D ≤ 3 months, *n* = 32; and **(e)** T1D > 3 months, *n* = 40. Each row is an individual subject with CDI calculated from the mean of unstimulated proliferation in triplicate experiments. **(f)** Median CDI for islet peptides.

We recruited five groups of consenting paediatric and adult participants (Table 1), including T1D ≤ 3 months, T1D > 3 months, healthy controls, islet antibody (AB)‐negative first‐degree relatives (FDR) without T1D and AB‐positive FDR with at least one AB and no clinical features of T1D (Table 1, Supplementary table 1). Using our previously optimised CFSE assay for proinsulin (PI)‐specific CD4^+^ T‐cell proliferative responses, we compared cell division indices (CDIs, see Methods) for multiple PI epitopes across the 5 groups. The CDIs for each PI epitope were significantly greater in participants recently diagnosed with diabetes (T1D ≤ 3 months) than either healthy controls or participants with T1D > 3 months (Figure 1). For both PI_33‐63_ and PI_48‐62_, CDIs were significantly higher in AB‐positive pre‐diabetic FDR than in healthy controls. The CDI for PI_33‐63_ was also greater in AB‐positive children than in participants with T1D > 3 months. For PI_48‐62_, CDIs were significantly higher in AB‐negative pre‐diabetic FDR than in healthy controls, and a similar trend was observed for PI_33‐63_. The overall trend towards higher responses to hEL:A‐chain, hEGGG:C‐peptide and hEGGG:IAPP2 in participants with T1D ≤ 3 months and AB‐positive FDR compared to healthy controls and T1D > 3 months was similar to that of PI_33‐63_, but there was greater heterogeneity in the response (Figure 2). Consistent with this, CDIs for hEL:A‐chain, hEGGG:C‐peptide and hEGGG:IAPP2 correlated with CDIs for PI_33‐63_ (T1D ≤ 3 months *r* ^2^ = 0.38 *P* = 0.027; *r*_s_ = 0.64 *P* = 0.003; *r*_s_ = 0.40 *P* = 0.035, respectively).

To determine the frequency of positive responses, we set CDI ≥ 3.0 as the threshold based on a specificity of 81% for PI_33‐63_‐specific CD4^+^ T‐cell responses using the receiver‐operator characteristic curve analysis of T1D ≤ 3 months vs healthy controls (Supplementary table 2). These findings are consistent with a recent report.^25^ A CD4^+^ T‐cell response with CDI ≥ 3.0 to any epitope was detected in 91% (29/32) of participants with T1D ≤ 3 months, 82% (14/17) of AB‐positive FDR, 78% (7/9) of AB‐negative FDR, 35% (14/40) of T1D onset > 3 months and 31.35% (5/16) of healthy controls (Table 2).

**Table 1:** Baseline demographics

	Healthy Controls	AB‐Negative	AB‐Positive	T1D ≤ 3 months	T1D > 3 months
Number of subjects (children; adults)	16	9	17	32 (28;4)	40 (26;14)
Age in years: mean, range (children; adults)	28.5, 11–51**** ^,^ ^#^	11.6, 7–16**** ^,^ ^#^	15.8, 7‐27**** ^,^ ^#^	10.75, 2–26 (9.3, 2–16; 20.75, 18–26)	15.2, 4–56 (8.3, 4–16; 23.1, 17–56)
Gender female: male (children, adults)	6:6	2:7	7:10	16:12, 1:3	14:11, 7:1
Hba1c %: mean, range (children; adults)	5.2, 4.9–5.7**** ^,^ ^#^	5.0, 4.9–5.3**** ^,^ ^#^	5.1, 4.8–5.7**** ^,^ ^#^	6.85, 5.5–8.7*** ^,^ ^†^ (6.8, 5.5–8.7; 7.25, 5.9–8)	8.2, 5.5–12 (8.2, 5.5–11.6; 8.2, 6–12)
Duration of T1D, months: mean, range (children, adults)				0.68, 0–3**** ^,^ ^†^ (0.57, 0–3; 1.5, 0–2)	59.5, 5–229 (34.8, 5–136; 104.9, 26–229)
Estimated c‐peptide: mean ± sem (children)				0.67 ± 0.1^**,^ ^†^	0.19 ± 0.1

^#^ Compares to all T1D.

^†^ compares to T1D > 3 months.

* *P* < 0.05.

*** *P* < 0.001.

**** *P* < 0.0001 unpaired *t* test.

### PI_33‐63_‐specific responses are more frequent than responses to any other epitope containing PI sequences

**Figure 3:**
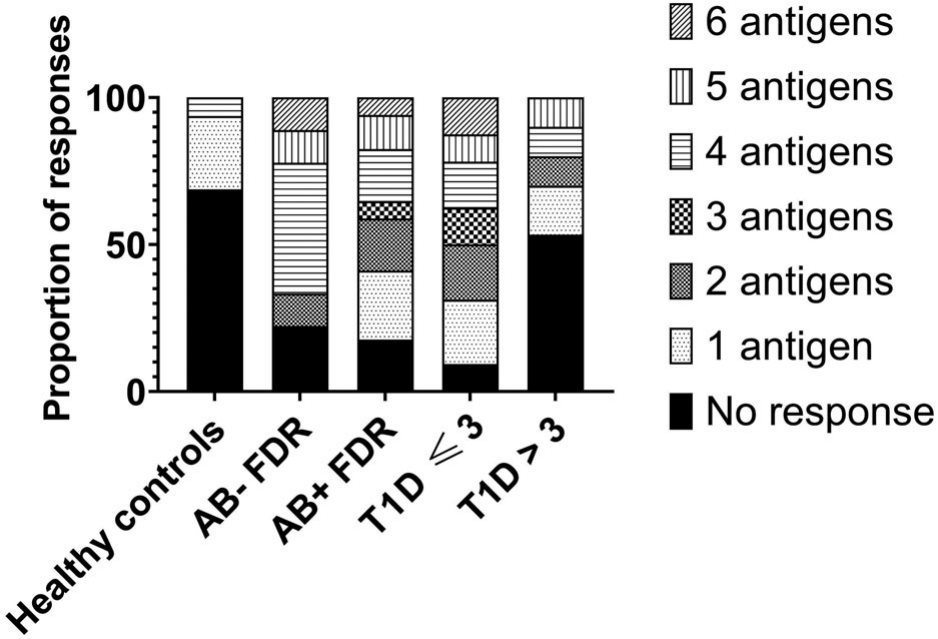
Response to multiple epitopes is greatest before and immediately after onset of T1D. Each bar depicts the proportion of subjects with responses (CDI > 3) to one or more proinsulin or HIP epitopes. CDI was calculated from the mean of unstimulated proliferation in triplicate experiments. Healthy control, *n* = 16; AB‐negative FDR, *n* = 9; AB‐positive FDR, *n* = 17; T1D ≤ 3 months, *n* = 32; T1D > 3 months, *n* = 40.

Of all the islet peptides, we observed CD4^+^ T‐cell proliferation most frequently in response to PI_33‐63_ across all groups (Table 2). We observed a consistently high median CDI to PI_33‐63_ and PI_48‐62_ in AB‐negative and AB‐positive FDR and participants with recent‐onset T1D (Figure 2, Table 2). In 69% (22/32) of these participants with T1D ≤ 3 months, CD4^+^ T cells responded to multiple epitopes. We also observed frequent CD4^+^ T‐cell proliferation to multiple epitopes in AB‐positive and AB‐negative FDR (Figure 3).

In view of the increase in PI_33‐63_‐specific CD4^+^ T‐cell proliferative responses across pre‐diabetic FDR, we assessed whether CDI increased as T1D risk increased with number of AB. There was no correlation between PI_33‐63_ CDI and the number of AB present (Supplementary figure 1), indicating that PI_33‐63_ CDI is an early marker of autoreactivity that does not increase with the development of islet AB.

### PI_33‐63_‐specific CD4^+^ T‐cell response correlates with estimated C‐peptide

**Figure 4:**
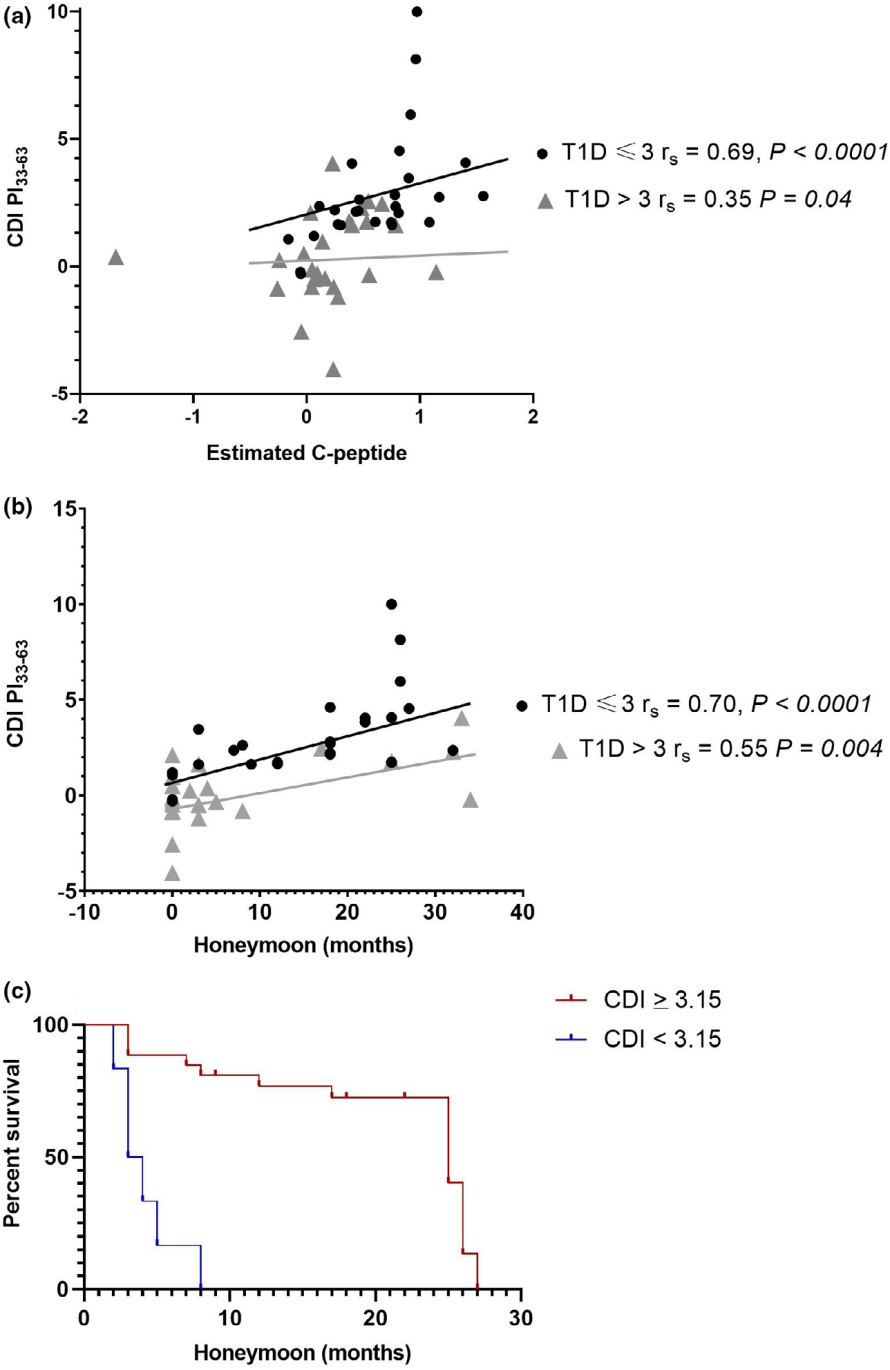
The response to PI_33‐63_ correlates with current and future β‐cell function. Correlation of CDI for PI_33‐63_ with **(a)** estimated C‐peptide at the same time point, and **(b)** duration of honeymoon (months). Each point represents an individual response with CDI calculated from the mean of unstimulated proliferation in triplicate experiments. **(c)** Survival analysis for all T1D patients based on CDI ≥ 3.15 discriminating T1D ≤ 3 months and T1D > 3 months, and where the honeymoon period was ≤ 30 months, *P* = 0.00624. T1D ≤ 3 months, *n* = 28; T1D > 3 months, *n* = 25.

In paediatric patients, C‐peptide can be estimated based on clinical parameters of age, gender, BMI‐Z score, HbA1c, time since diagnosis and insulin dose (see Methods).^3^ PI_33‐63_ CDI positively associated with estimated C‐peptide measured at the same time‐point (T1D ≤ 3 months *r*_s_ = 0.69, *P < *0.0001; T1D > 3 months, *r*_s_ = 0.35 *P = *0.04, Figure 4a). Furthermore, PI_33‐63_ CDI positively associated with the duration of the honeymoon (T1D ≤ 3 months *r*_s_ = 0.70, *P < *0.0001; T1D > 3 months, *r*_s_ = 0.55 *P = *0.004, Figure 4b) and predicted survival in honeymoon (*P = *0.00624, Figure 4c). Similar positive associations were found for PI_48‐62_ and PI_40‐52_ but not for the HIP‐specific peptides. PI_33‐63_ CDI was not associated with age (Supplementary figure 1) but was associated with the number of months since diagnosis (Supplementary figure 2). PI_33‐63_ CDI was not associated with insulin antibody status in AB‐positive FDR (Supplementary figure 3) or those with T1D (pre‐insulin treatment insulin antibody data was available for 38/72 participants with T1D).

### PI_33‐63_‐specific CD4^+^ T‐cell responses are more frequent in at‐risk or diabetic subjects with HLA‐DR3‐DQ8 or HLA‐DR4‐DQ2

**Figure 5:**
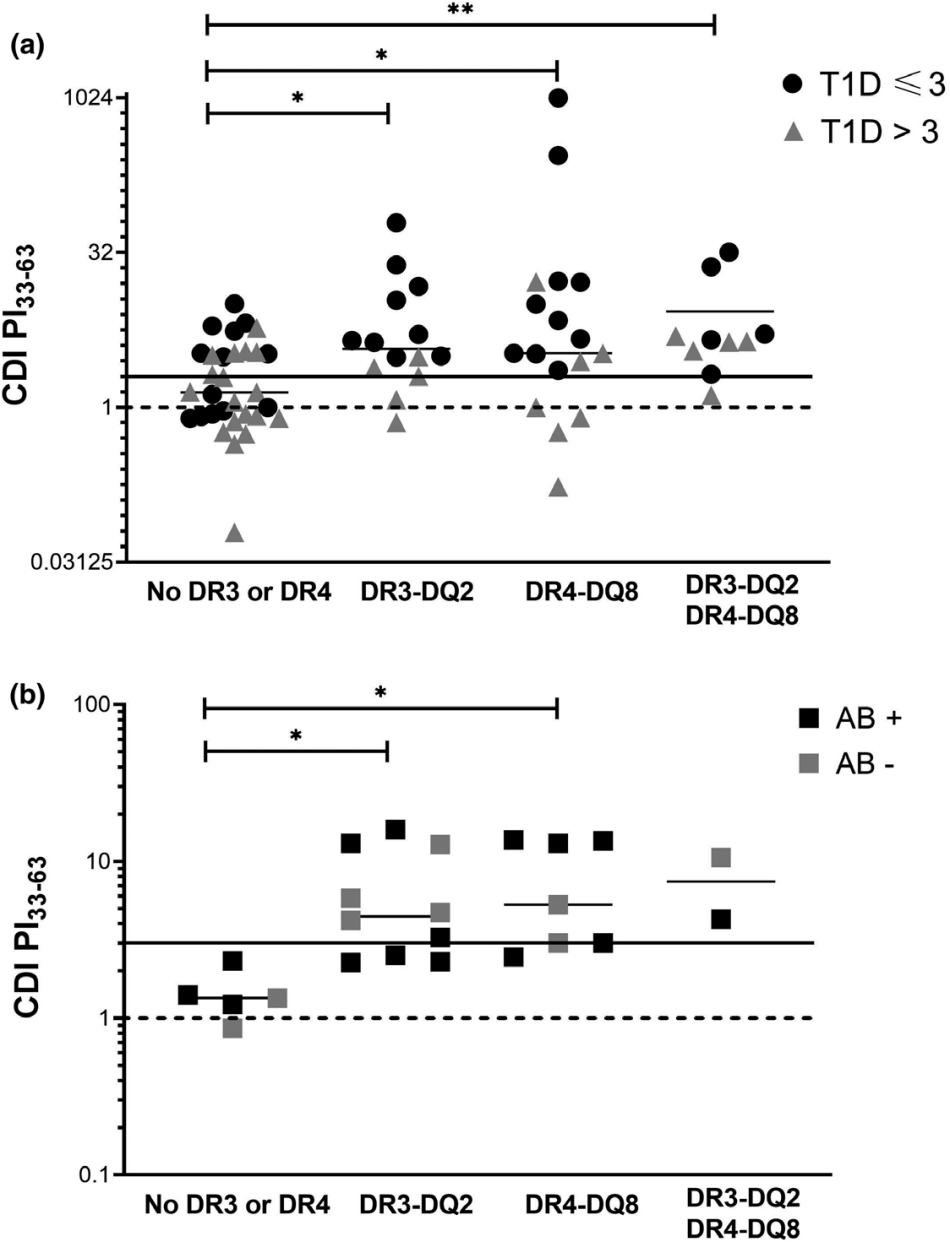
PI_33‐63_ responses in individuals with HLA‐DR3‐DQ2 and/or HLA‐DR4‐DR8. **(a)** All T1D **(b)** AB‐negative FDR and AB‐positive FDR. Each point represents an individual subject with CDI calculated from the mean of unstimulated proliferation in triplicate experiments. Solid line, CDI = 3. Dashed line, CDI = 1. AB‐negative FDR, *n* = 9; AB‐positive FDR, *n* = 15; T1D ≤ 3 months, *n* = 32; T1D > 3 months, *n* = 40.

PI_33‐63_ CDIs from all (52/52) participants with T1D were grouped into high‐risk HLA‐DR3‐DQ2, HLA DR4‐DQ8, highest‐risk HLA DR3‐DQ2 and DR4‐DQ8 or lower‐risk non‐HLA‐DR3/DR4. PI_33‐63_ CDIs were significantly higher among T1D participants with any high‐risk allele or allele combination than no high‐risk allele (Figure 5a). A similar pattern was observed among the combined AB‐positive (9/9) and AB‐negative FDR (15/17), although the analysis was limited by small numbers of subjects in each group (Figure 5b). Comparable trends were observed between CDI for PI_48‐62_ and PI_40‐52_ and high‐risk alleles for T1D and FDR. No HLA associations were evident for the HIP‐specific CD4^+^ T‐cell responses.

## Discussion

Characterisation of islet antigen‐specific CD4^+^ and CD8^+^ T cells during the prodrome and after onset of T1D is crucial, in order to understand their role in disease pathogenesis, to relate T‐cell responses to treatment outcomes, to identify subjects potentially suited to immunotherapy and to characterise T‐cell responses to antigen‐specific immunotherapies. However, a robust T‐cell biomarker that tracks immunological correlates of β‐cell function has remained an unrealised goal. Using a previously optimised assay, we show that proinsulin epitope‐specific proliferative responses were most frequent towards PI_33‐63_ in children and adults with T1D and in individuals at risk, and that PI_33‐63_‐specific responses were higher in individuals with HLA class II T1D‐associated risk alleles than in individuals without these risk alleles. While high levels of PI epitope‐specific PB CD4^+^ T‐cell proliferation were evident in AB− and AB+ at‐risk FDR and within 3 months of T1D diagnosis relative to healthy controls carrying HLA risk alleles, they declined in individuals with T1D for longer than 3 months. These data indicate that PI_33‐63_‐specific CD4^+^ T‐cell proliferation is a marker of active islet autoimmunity, which is demonstrable in FDR at genetic risk of T1D even before the development of islet AB. Furthermore, after T1D diagnosis, PI_33‐63_‐specific PB CD4^+^ T‐cell proliferation correlates with estimated C‐peptide and predicts survival in partial remission. Thus, in children diagnosed with T1D, high PI_33‐63_‐specific CD4^+^ T‐cell responses identify residual predicted endogenous β‐cell mass and predict a better 2‐year survival in partial remission.

Previous studies also identified antigen‐specific CD4^+^ T cells in PB^25^, ^32^, ^33^, ^34^, ^35^ and in the islets of organ donors with T1D, including multiple islet antigens and HIPs.^11^, ^30^ This study extends that work and demonstrates that proinsulin immune reactivity is best captured by assays incorporating the PI_33‐63_ full‐length C‐peptide sequence, which contains multiple epitopes,^25^, ^32^, ^36^ potentially increasing the pool of antigen‐reactive T cells. Shorter epitopes within PI_33‐63_ are presented by several HLA‐DR and HLA‐DQ molecules, thus generating T‐cell responses from individuals with varied HLA types.^25^

Unlike PI‐specific immunogenicity, which was common across the groups tested, T‐cell autoreactivity to HIP neoepitopes^12^ was more heterogeneous. Emerging evidence suggests that the generation of neoepitopes is linked to β‐cell stress and activates T cells, amplifying the autoreactive repertoire. However, the presence of HIPs within the islets does not appear sufficient to cause disease, as HIPs have been found in islets of donors without T1D.^37^ Heterogeneity in the immune response to HIPs may relate to different levels of β‐cell stress, HLA genotype,^38^ and the timing and extent of HIP formation *in vivo*. In NOD mice, PB T cells recognising HIPs were detected with increasing frequency as they progressed towards diabetes.^39^ While we did not undertake longitudinal studies, HIP‐specific T‐cell reactivity correlated with PI‐specific reactivity, indicating that epitope spreading may be related to the magnitude of the autoimmune response. In participants with T1D ≤ 3 months, AB‐negative and AB‐positive FDR, the increased islet‐specific CD4^+^ T‐cell autoimmunity presumably indicates ongoing priming of immune cells and epitope spreading to multiple β‐cell antigens prior to, or soon after the clinical manifestation of disease. Future longitudinal studies could map changes and potential epitope spreading in autoantigen‐specific CD4^+^ T‐cell responses before and after the onset of T1D.

We showed that the level of PI_33‐63_‐specific T‐cell proliferation was associated with concurrent β‐cell function and predicted survival in partial remission. Furthermore, proliferation of PI_33‐63_‐specific T cells correlated with proliferation to HIPs. If these islet‐specific T cells contribute to β‐cell destruction, this observation is unexpected. The association with concomitant β‐cell function may reflect a larger β‐cell mass and thus antigen availability to sustain antigen‐specific CD4^+^ T‐cell memory, and potential for clonal expansion upon peptide re‐exposure *in vitro*.^40^ However, the association of PI_33‐63_‐specific proliferating T cells with future honeymoon duration suggests they may have regulatory function, with capacity to delay disease progression. Proliferating cells produce IL‐2, which is needed to support CD25^+^ regulatory T cells, including Foxp3^+^CD25^hi^CD127^lo^ and CD25^+^CD127^hi^ T cells. The frequency of CD25^+^CD127^hi^ T cells was also recently shown to predict survival in honeymoon.^41^ Polymorphisms in multiple genes in the IL‐2 pathway can predispose to T1D,^42^, ^43^, ^44^ and defects in IL‐2 receptor signalling reduce Foxp3 expression in CD25^+^ regulatory T cells in T1D patients, prompting trials of both low‐dose IL‐2 and Treg cell therapy.^45^, ^46^, ^47^

Autoimmune β‐cell damage occurs before the onset of clinically apparent disease, and PI_33‐63_ CDI may be a useful biomarker of autoimmunity in people at genetic risk for T1D, even in the absence of islet AB. We show that almost all FDR responded to at least one of the proinsulin epitopes. Since many of these individuals are unlikely to develop T1D, this observation also supports the hypothesis that the autoantigen‐specific CD4^+^ T cells may have regulatory function. In a recent analysis of PB cytokine responses to a partially overlapping set of islet epitopes (PI_40‐52_, hEL:A‐chain and hEGGG:IAPP2) using ELISPOT in FDR, the IFNg/IL‐10 ratio in response to certain HIPs correlated with clinical parameters of progression to T1D, including islet AB titres and evidence of glucose intolerance.^48^ Given that HIP‐specific proliferative responses correlated with PI_33‐63_‐specific responses and that a PI_33‐63_ CDI ≥ 3 predicted survival in honeymoon after T1D onset, it will be of interest to determine whether PI_33‐63_ CDI also stratifies the risk of developing T1D, adding new insights into the classification of presymptomatic T1D.^49^ Further work is also needed to characterise the phenotype and function of the proliferating PI_33‐63_‐specific CD4^+^ T cells, and to map changes in progressors and non‐progressors to T1D, and in individuals with sustained remission in clinical trials of immunotherapy.

Although the presence of islet antibodies is associated with the risk of developing T1D,^15^ their predictive role after disease onset is not well understood. An inverse correlation between humoral and cellular islet autoimmunity has been previously described.^50^ PI‐specific CDI did not correlate with the number of autoantibody specificities in AB‐positive siblings. Furthermore, both AB‐negative and AB‐positive siblings had PI‐specific CDI, confirming that the presence of AB was not necessary for islet peptide‐specific T‐cell proliferative responses.

A key strength of this study is that we quantified PI‐specific responses in PB using an optimised, fit‐for‐purpose assay, validated for intra‐assay and inter‐assay repeatability.^31^ However, our study has limitations. While the at‐risk and T1D participant groups comprised adults and children, the healthy control group was significantly older. We limited the pool of peptides tested to those derived from proinsulin, based on previous publications. However, other published epitopes^51^, ^52^ within the proinsulin A chain, B chain and other islet antigens such as GAD‐65 or ZnT8 could also be informative, particularly as many AB‐positive siblings developed antibodies against GAD‐65 and ZnT8 (Table 2). Using ELISPOT assays to measure IFN‐γ and IL‐10 CD4^+^ T‐cell responses to a variety of islet epitopes, a previous study found fewer adults than children with recent‐onset T1D made IFN‐γ responses.^24^ While we found no relationship between CDI and age, we did not assess cytokine production in the current studies. Furthermore, although our cohort had sufficient power to determine the relationships of CDI to clinical features and HLA, it was not large enough to fully explore the immunological heterogeneity of T1D. Validation of these findings in additional cohorts would add value and may reveal interactions between variables.

**Table 2:** The proportion of participants responding to full‐length C‐peptide (PI_33‐63_) is higher than for any other proinsulin or HIP epitope.

Epitope	Healthy controls (%)	AB‐negative (%)	AB‐positive (%)	T1D ≤ 3 months (%)	T1D > 3 months (%)
PI_33‐63_	4/16 (25%)^#^ ^,^ ****	7/9 (78%)	10/17 (58%)	28/32 (88%)	13/40 (33%)^#^ ^,^ ****
PI_48‐62_	1/14 (7%)^#^ ^,^ *	6/9 (75%)	9/17 (53%)	13/29 (45%)	9/37 (24%)
PI_40‐52_	0/13 (0%)^#^ ^,^ ***	6/8 (75%)	3/17 (18%)	12/28 (43%)	8/38 (21%)
hEL:A‐chain	1/14 (7%)^#^ ^,^ *	4/8 (50%)	7/17 (41%)	13/28 (46%)	10/38 (26%)
hEGGG:C‐pep	0/14 (0%)^#^ ^,^ ***	3/8 (38%)	4/17 (24%)	12/26 (46%)	10/35 (29%)
hEGGG:IAPP2	2/14 (14%)^#^ ^,^ *	3/8 (38%)	3/17 (18%)	12/25 (48%)	8/37 (22%)

The percentage of each group with CDI ≥ 3 to each auto‐antigenic epitope. The denominator indicates the number of participant samples in each group for which results are available with CDI calculated from the mean of unstimulated proliferation in triplicate experiments.

^#^ Comparison with T1D ≤ 3 months.

* *P* < 0.05.

*** *P* < 0.001.

**** *P* < 0.0001, Fisher’s exact test.

In summary, CD4^+^ T‐cell proliferative responses to proinsulin‐containing autoantigens are frequently detectable prior to and immediately after T1D onset, followed by a decline. Proinsulin_33‐63_‐specific CD4^+^ T‐cell response is a novel marker of current and future endogenous β‐cell function.

## METHODS

### Subjects

T1D was defined according to the criteria from the American Diabetes Association.^53^ Healthy controls were locally collected participants or Australian Bone Marrow Donor Registry participants carrying either HLA‐DQ2 or HLA‐DQ8 alleles but with no personal or family history of T1D and without T1D‐related antibodies. The absence of AB against insulin, glutamic acid decarboxylase 65 (GAD65) and islet antigen insulinoma‐associated protein‐2 (IA‐2) was confirmed in all healthy controls and AB‐negative FDR. Subjects were excluded if they were receiving immune‐modulatory drugs or had other autoimmune disease except for patients with hypothyroidism receiving appropriate thyroid hormone replacement or coeliac disease on gluten exclusion. At the time of sample collection, adequately treated hypothyroidism was present in one patient with T1D ≤ 3 months (1/32). Three participants with T1D > 3 months (3/40) had both hypothyroidism on appropriate replacement and coeliac disease on gluten exclusion. The study was approved by the Children’s Health Queensland, Mater Hospital, Australian Red Cross and University of Queensland Human Research Ethics Committees. No blinding or randomisation was used in this study. All HLA typing was performed by the Australian Red Cross Blood Services.

### Estimate of β‐cell function

In paediatric patients with T1D, we estimated endogenous C‐peptide levels according to a previously reported method.^3^ The estimated C‐peptide model incorporates age, gender, BMI‐Z score, HbA1c, time since diagnosis and insulin dose. Based on this model, an estimated C‐peptide of 0.4 correlates with a stimulated C‐peptide of 0.3 nmol L^−1^. We estimated C‐peptide levels at 3‐month intervals over 2 years. Paediatric patients were defined as being in partial remission (honeymoon) if their estimated C‐peptide measured ≥ 0.4.

### Antigens

Tetanus toxoid (AJ Vaccines, 10 LfU mL^−1^), ultra‐LEAF‐purified anti‐human CD3 (Biolegend, San Diego USA, Clone OKT3, 0.1 µg mL^−1^) and proinsulin (PI) or HIP epitopes (GL Biochem, Shanghai, China) were included in CD4^+^ T‐cell proliferation assays at a final concentration of 10 µM.^31^ Proinsulin epitopes included the following sequences: PI_33‐63_ (C‐peptide, Sequence: EAEDLQVGQVELGGGPGAGSLQPLALEGSLQ), PI_48‐62_ (PI_48‐62_, Sequence: PGAGSLQPLALEGSL) and PI_40‐52_ (PI_40‐52_, Sequence: GQVELGGGPGAGS). HIPs containing parts of the PI_33‐63_ sequence included the following: hEGGG:C‐peptide (Sequence: GQVELGGGEAEDLQV), hEL:A‐chain (Sequence: SLQPLALGIVEQCC) and hEGGG:IAPP2 (Sequence: GQVELGGGNAVEVLK).

### Cell preparation, CFSE Staining and T‐cell stimulation

Peripheral blood mononuclear cells (PBMC) were freshly isolated and labelled with carboxyfluorescein diacetate succinimidyl ester (CFSE, CellTrace™ CFSE Cell Proliferation Kit for flow cytometry, Invitrogen, Thermo Fisher Scientific, Waltham, Massachusetts, USA) as described.^31^, ^54^ CFSE‐stained cells (2 × 10^6^ PBMC mL^−1^, 200 μL per well) were cultured for 7 days with medium alone (negative control), peptides or with positive controls, tetanus toxoid and/or anti‐CD3 to obtain a cell division index (CDI).^54^ Three replicates were tested for each experiment. The CDI is the ratio of CD4^+^T cells that proliferated in response to antigen, relative to cells that proliferated in the absence of antigen. The CDI was calculated based on a fixed number of 5000 CD4^+^ CFSE^(undivided)^ cells using the following formula:

$$\text{CDI}=\frac{\text{number}\;\text{of}\;\text{divided}\;\text{CD}4^{+}T\;\text{cells}\;\text{per}\;5,000\;\text{CD}4^{+}T\;\text{cells}\;\text{in}\;\text{CFSE}\;(\text{undivided})\;{\text{from}}^{‘}\text{with}\;{\text{antigen}}^{\text{’}}\;\text{group}}{\text{number}\;\text{of}\;\text{divided}\;\text{CD}4^{+}T\;\text{cells}\;\text{per}\;5,000\;\text{CD}4^{+}T\;\text{cells}\;\text{in}\;\text{CFSE}\;(\text{undivided})\;{\text{from}}^{‘}\text{without}\;{\text{antigen}}^{\text{’}}\;\text{group}}\;$$

As the assay was run in triplicate, the mean of unstimulated proliferation was used to calculate the CDI. A CDI of ≥ 3 was determined to represent the threshold for the positive control responses. Data from assays in which the CDI for the positive controls, tetanus toxoid and anti‐CD3, did not exceed 3.0 were excluded from the analysis.

### Statistical analysis

Pairwise comparison between categorical variables was conducted with the Chi‐square test, or the Fisher’s exact test if one or more cells in the contingency table had an expected frequency of ≤ 5 using Graphpad Prism (Version 7, San Diego, CA, USA) and R Statistical Software (Version 3.5.3, Foundation for Statistical Computing, Vienna, Austria). The Kruskal–Wallis test was used for multiple comparisons, followed by post‐hoc Dunn’s test, and *P‐*values were adjusted with the Benjamini–Hochberg method. Comparisons between group data were made using the paired two‐tailed *t*‐test. Statistical significance was defined as *P* < 0.05. The Kaplan–Meier survival analysis was performed with the R package *Survival* (https://cran.r‐project.org/web/packages/survival/index.html). Patients were assigned to two groups high score (≥ median PI_33‐63_ CDI of 3.15) and low score (< median PI_33‐63_ CDI of 3.15). A Kaplan–Meier survival using the default setting extracted a *P*‐value for a honeymoon period of up to 30 months.

## Conflict of interest

We have no conflict of interest to declare.

## Author contribution

**Yassmin Mansela Musthaffa:** Conceptualization; Data curation; Formal analysis; Funding acquisition; Investigation; Methodology; Resources; Software; Validation; Visualization; Writing‐original draft; Writing‐review & editing. **Emma Estelle Hamilton‐Williams:** Conceptualization; Supervision; Validation; Writing‐original draft; Writing‐review & editing. **Hanno Nel:** Data curation; Investigation. **Anne‐Sophie Bergot:** Investigation. **Ahmed Mehdi:** Formal analysis; Writing‐review & editing. **Mark Harris:** Conceptualization; Methodology; Supervision; Validation; Visualization; Writing‐original draft; Writing‐review & editing. **Ranjeny Thomas:** Conceptualization; Formal analysis; Funding acquisition; Project administration; Resources; Supervision; Validation; Visualization; Writing‐original draft; Writing‐review & editing.
