# Supplementary material for: Proinsulin‐specific T‐cell responses correlate with estimated c‐peptide and predict partial remission duration in type 1 diabetes
Source: Clin Transl Immunology. 2021 Jul 26;10(7):e1315. doi: 10.1002/cti2.1315 (PMC8312239; doi:10.1002/cti2.1315)
Supplement: Supplementary file 1 [file CTI2-10-e1315-s001.pdf]

**TITLE:** Proinsulin-specific t-cell responses correlate with estimated c-peptide and predict partial remission duration in type 1 diabetes

**RUNNING TITLE:** Proinsulin-specific T-cells as a T1D biomarker

## SUPPLEMENTARY FIGURES AND TABLES

**Table 1. Islet antibody status of participants**

| Islet antibody | Healthy controls (16) | AB-negative FDR (9) | AB-positive FDR (17) |
|---|---|---|---|
| Glutamic acid decarboxylase 65 (GAD 65) | 0 | 0 | 12 (71%) |
| Islet tyrosine phosphatase-like protein (IA-2) | 0 | 0 | 9 (53%) |
| Microinsulin (miaa) | 0 | 0 | 15 (88%) |
| Islet cell (ICA) | 0 | 0 | 12 (71%) |
| Zinc transporter 8 (ZnT8) | 0 | 0 | 9 (53%) |
| >1 islet antibody | 0 | 0 | 11 (64%) |

**Supplementary Table 2. ROC, Receiver Operator Characteristic Curve analysis of T1D ≤ 3 months (n=22) and Healthy controls (n=12).** CDI, Cell Division Index is calculated from the mean of unstimulated proliferation in triplicate experiments.

| **Cell-Division Index** | **Sensitivity (%)** | **Specificity (%)** |
|---|---|---|
| **> 2.0** | 91% | 73% |
| **> 2.6** | 81% | 73% |
| **> 2.9** | 77% | 73% |
| **> 3.0** | 77% | 81% |
| **> 3.5** | 63% | 81% |

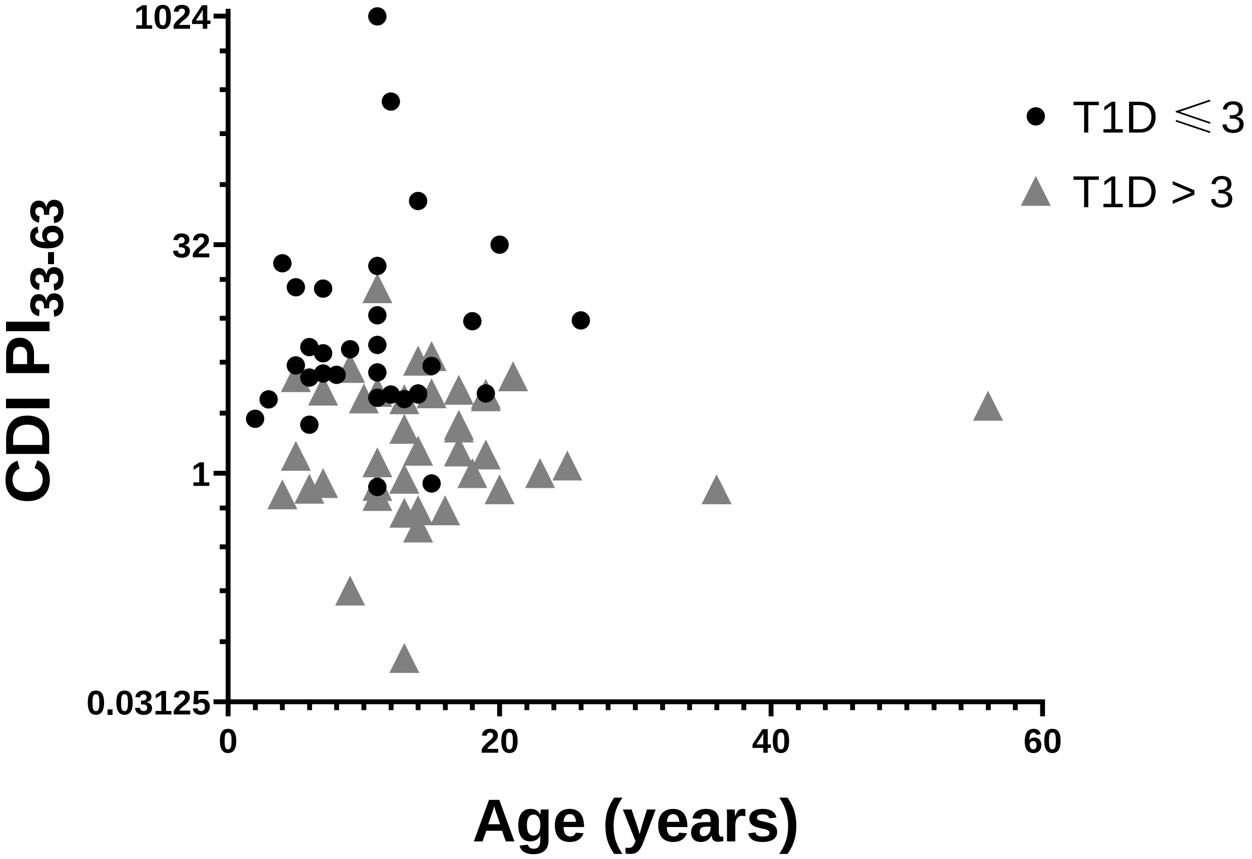


**Supplementary Figure 1. CD4⁺ T-cell proliferative responses to proinsulin$_{33\text{-}63}$ relative to age for T1D subjects. ($r_s$ = -0.18, P- = 0.11).** CDI was calculated from the mean of unstimulated proliferation in triplicate experiments. T1D ≤ 3 months, n= 32; T1D > 3 months, n = 40.

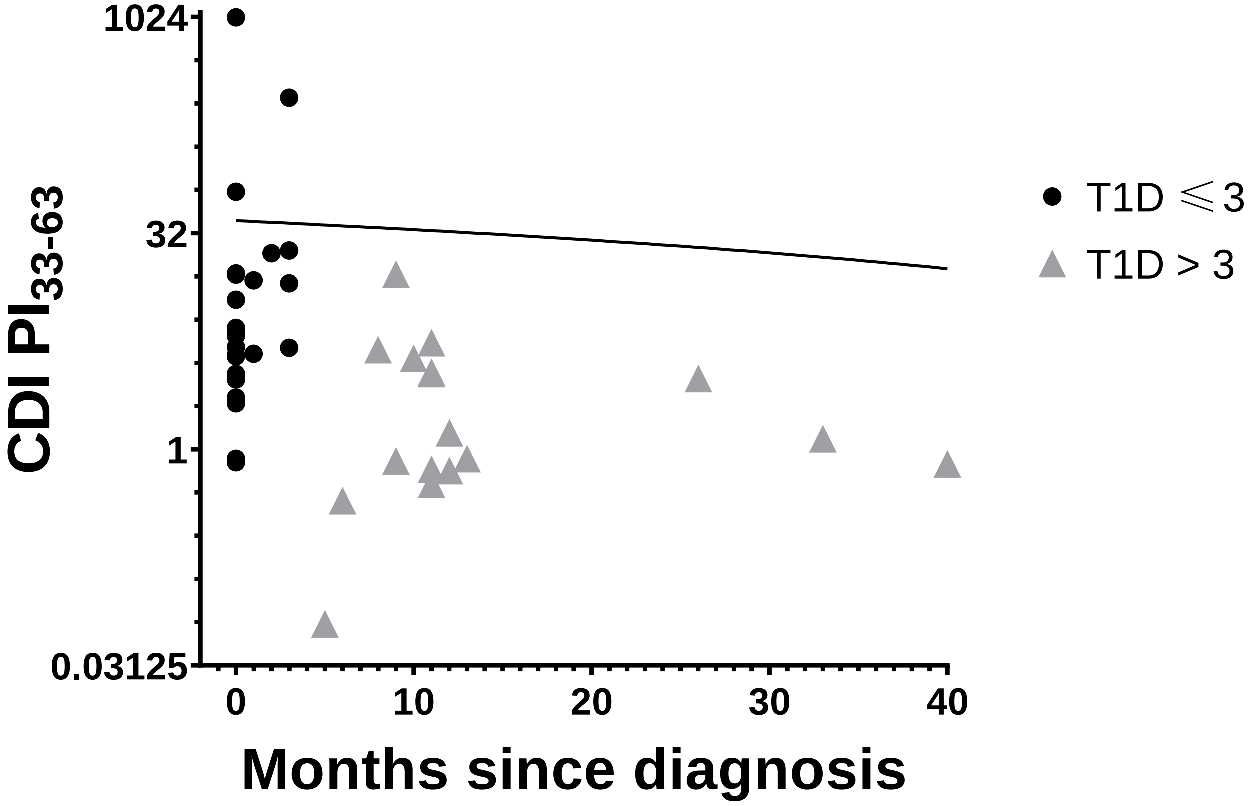


**Supplementary Figure 2. CD4+ T cell proliferative responses to proinsulin$_{33\text{-}63}$ relative to months since diagnosis for T1D subjects. ($r_s$ = -0.47, P- = 0.0005).**

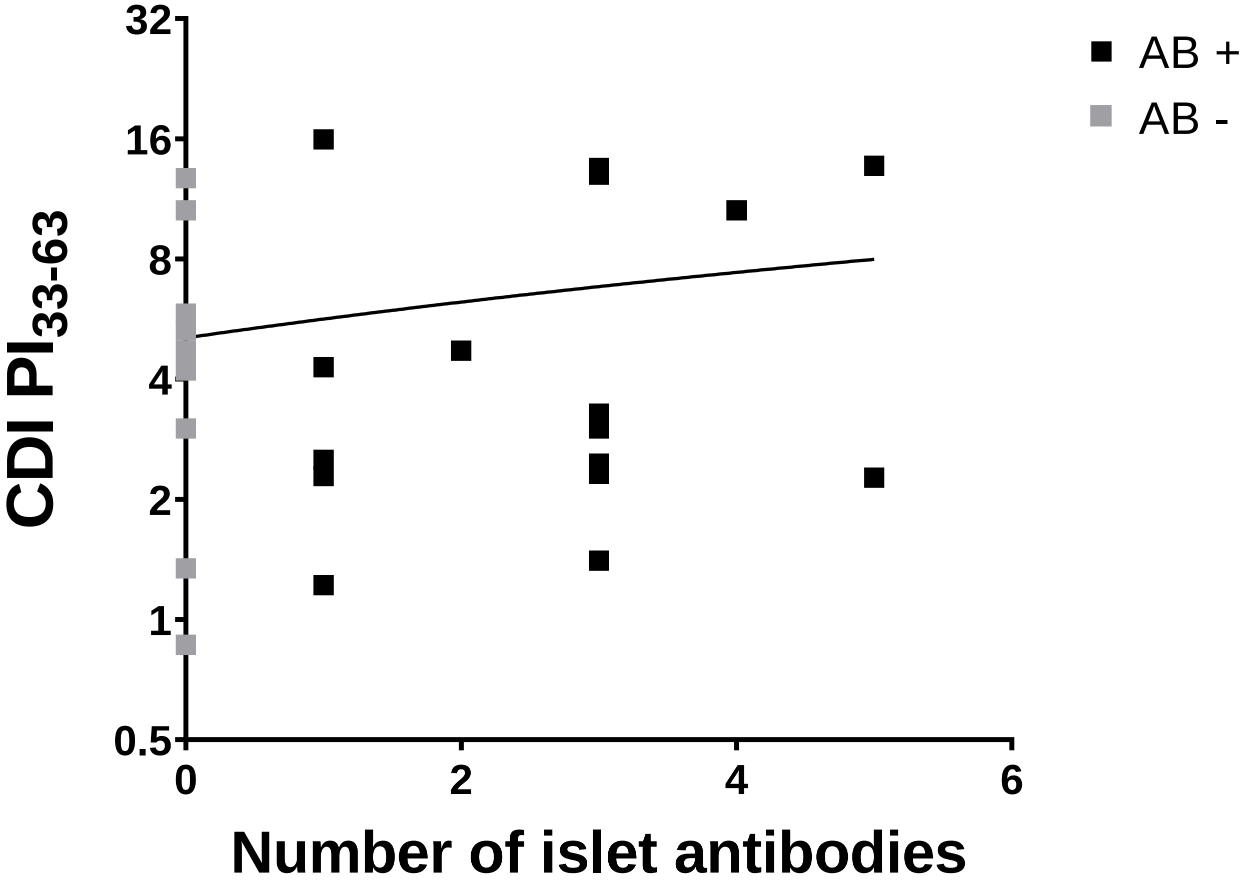


**Supplementary Figure 3. CD4+ T cell proliferative responses to $proinsulin_{33-63}$ relative to number of islet antibodies for prediabetes subjects. (r2 =0.14, P- = 0.58).**
